# Diversity and Characterization of Multicellular Magnetotactic Prokaryotes From Coral Reef Habitats of the Paracel Islands, South China Sea

**DOI:** 10.3389/fmicb.2018.02135

**Published:** 2018-09-11

**Authors:** Zhaojie Teng, Yuyang Zhang, Wenyan Zhang, Hongmiao Pan, Jianhong Xu, Hui Huang, Tian Xiao, Long-Fei Wu

**Affiliations:** ^1^CAS Key Laboratory of Marine Ecology and Environmental Sciences, Institute of Oceanology, Chinese Academy of Sciences, Qingdao, China; ^2^Laboratory for Marine Ecology and Environmental Science, Qingdao National Laboratory for Marine Science and Technology, Qingdao, China; ^3^Center for Ocean Mega-Science, Chinese Academy of Sciences, Qingdao, China; ^4^Key Laboratory of Marine Bio-resources Sustainable Utilization, South China Sea Institute of Oceanology, Chinese Academy of Sciences, Guangzhou, China; ^5^University of Chinese Academy of Sciences, Beijing, China; ^6^Aix Marseille University, CNRS, LCB, Marseille, France; ^7^International Associated Laboratory of Evolution and Development of Magnetotactic Multicellular Organisms (LIA-MagMC), CNRS-CAS, Qingdao, China

**Keywords:** multicellular magnetotactic prokaryotes (MMPs), diversity, phylogenetic analysis, coral reef, South China Sea

## Abstract

While multicellular magnetotactic prokaryotes (MMPs) are ubiquitous in marine environments, the diversity of MMPs in sediments of coral reef ecosystems has rarely been reported. In this study, we made an investigation on the diversity and characteristics of MMPs in sediments at 11 stations in coral reef habitats of the Paracel Islands. The results showed that MMPs were present at nine stations, with spherical mulberry-like MMPs (s-MMPs) found at all stations and ellipsoidal pineapple-like MMPs (e-MMPs) found at seven stations. The maximum abundance of MMPs was 6 ind./cm^3^. Phylogenetic analysis revealed the presence of one e-MMP species and five s-MMP species including two species of a new genus. The results indicate that coral reef habitats of the Paracel Islands have a high diversity of MMPs that bio-mineralize multiple intracellular chains of iron crystals and play important role in iron cycling in such oligotrophic environment. These observations provide new perspective of the diversity of MMPs in general and expand knowledge of the occurrence of MMPs in coral reef habitats.

## Introduction

Magnetotactic bacteria (MTB) are a morphologically, phylogenetically, and metabolically diverse group of prokaryotes (Lefèvre and Wu, 2013; Lefèvre et al., 2013; Araujo et al., 2015) that form intracellular magnetic nanoparticles composed of magnetite (Fe_3_O_4_) or greigite (Fe_3_S_4_) enveloped in a lipid bilayer within the cell (Bazylinski and Frankel, 2004; Edwards and Bazylinski, 2008; Araujo et al., 2015). Cell morphologies of MTB include cocci, rods, spirilla, vibrios, barbell-shaped, and multicellular forms (Blakemore, 1982; Bazylinski and Frankel, 2004). The multicellular forms are referred to as multicellular magnetotactic prokaryotes (MMPs) (Farina et al., 1983; Rodgers et al., 1990).

Two distinct morphotypes of MMPs have been observed, including spherical mulberry-like MMPs (s-MMPs) (Abreu et al., 2007; Simmons and Edwards, 2007; Wenter et al., 2009; Zhou et al., 2013; Zhang et al., 2014) and ellipsoidal pineapple-like MMPs (e-MMPs) (Lefèvre et al., 2007; Zhou et al., 2012; Chen et al., 2015, 2016). The s-MMPs are typically 3–12 μm in diameter and composed of 10–40 cells arranged with helical symmetry (Abreu et al., 2007; Keim et al., 2007; Zhou et al., 2011, 2013; Zhang et al., 2014). The e-MMPs are typically 8–23 μm in length and 7–17 μm in width, and consist of 28–101 cells arranged in interlaced cell circles (Lefèvre et al., 2007; Zhou et al., 2012; Chen et al., 2015, 2016). Both morphotypes of MMPs have peritrichous flagella (Keim et al., 2004a; Abreu et al., 2006, 2007; Zhang et al., 2014; Chen et al., 2015) and are capable of producing magnetite and/or greigite magnetosomes (Keim et al., 2004a; Zhou et al., 2011, 2012, 2013; Abreu et al., 2013; Zhang et al., 2014; Chen et al., 2015). Phylogenetic analysis based on 16S rRNA gene showed that both s-MMPs and e-MMPs are affiliated with the *Deltaproteobacteria* class (Delong et al., 1993; Keim et al., 2004b; Simmons et al., 2004; Simmons and Edwards, 2007). The s-MMPs consist of several clades, possibly forming numerous species (Farina et al., 1983; Abreu et al., 2007; Simmons and Edwards, 2007; Wenter et al., 2009; Zhou et al., 2012, 2013). The e-MMPs are closely related to s-MMPs, but appear to belong to different genera (Lefèvre et al., 2007; Zhou et al., 2011, 2012, 2013).

The s-MMPs morphotype was first described by Farina et al. (1983). They are cosmopolitan in various saline aquatic habitats, including coastal lagoons (Farina et al., 1983, 1990; Abreu et al., 2007, 2013), salt water lagoons (Pósfai et al., 1998; Simmons and Edwards, 2007) and lakes (Lefèvre et al., 2010), salt marshes (Delong et al., 1993; Pósfai et al., 1998; Simmons and Edwards, 2007; Edwards and Bazylinski, 2008; Wenter et al., 2009), and intertidal zones (Zhou et al., 2011, 2013; Zhang et al., 2014). In 2007, e-MMPs were first found from the sediments of the Mediterranean Sea (Lefèvre et al., 2007) and have now been reported from the Mediterranean Sea (France) (Lefèvre et al., 2007; Chen et al., 2016), the Yellow Sea (China) (Zhou et al., 2011, 2012, 2013; Zhang et al., 2014; Chen et al., 2015), and the intertidal zone of Drummond Island in the South China Sea (Chen et al., 2016). The abundance of two morphs of MMPs varies according to the sampling sites, depth and seasons, usually several hundred individuals per centimeter cube (ind./cm^3^) based on the investigation before (Abreu et al., 2007; Wenter et al., 2009; Zhou et al., 2012, 2013; Zhang et al., 2014; Chen et al., 2015, 2016). In general, the MMPs were commonly observed with a maximum abundance in summer (Lefèvre et al., 2007; Zhou et al., 2012, 2013; Chen et al., 2015). These findings support the paleoecology envision that warm periods are benefited to the growth of MTB, augmenting the concentration of fine-grained magnetite within the sediment (Lefèvre et al., 2007).

Over the last two decades, comprehensive information on the phylogeny, morphology, structure, swimming behavior, and genomes of MMPs has been obtained (Delong et al., 1993; Keim et al., 2004a,b; Simmons et al., 2004; Abreu et al., 2007; Lefèvre et al., 2007; Chen et al., 2015). The MMPs have attracted more attention than the unicellular MTB because of their complex motility and unique many-celled arrangement (Farina et al., 1983, 1990; Rodgers et al., 1990; Keim et al., 2004a, 2007; Zhou et al., 2012, 2013; De Azevedo et al., 2013; Chen et al., 2015).

The study of MMPs has focused mainly on marine ecosystems. Coral reefs are the most diverse of all marine ecosystems and are considered to be one of the most complexes. Coral reef systems have extremely high habitat heterogeneity and are important in maintaining enormous biological diversity (Moberg and Folke, 1999). Although coral reefs represent only a small fraction of the marine ecosystem (Guan et al., 2015), they are extremely important for nutrient cycling (Garren and Azam, 2012) in shallow, oligotrophic tropical waters (Moberg and Folke, 1999). Coral reef bacterial communities are key participants in the reef nutrient cycling and occupy a range of different habitats including sediments (Bourne and Webster, 2013). So far, only a single basic study of unicellular MTB from coral reefs, performed in the Gulf of Mannar (India), has been reported (Kannapiran et al., 1999). Consequently, more studies in coral reef habitats of the diversity and distribution of MTB, especially MMPs, are needed.

To expand knowledge of MMPs in coral reef habitats we investigated the biogeography and occurrence of these microbes in the Paracel Islands (South China Sea). We used microscopy and micromanipulation combined with whole genome amplification (WGA) to study the morphological characteristics and taxonomic diversity of MMPs from coral reef ecosystems of the Paracel Islands. Additionally, we discussed about the potential relationship between the physical and chemical characteristics and occurrence of MMPs. These results provide newly data for a better understanding of prevalence and abundance of MMPs in coral reef systems, may imply the cosmopolitism distribution of MMPs in these distinctive systems, and reveal the role of MMPs in coral reef habitats to some extent.

## Materials and Methods

### Sediment Sampling and Enrichment of MMPs

The Paracel Islands is a group of approximately 130 islets and reefs in the South China Sea approximately 320 km southeast of Hainan Provence; they are divided into the Xuande group in the northeast, and the Yongle group in the west (Ye, 1996; Wang et al., 2011). From 24 July to 4 August 2016, sediment and water samples were collected at 11 stations in coral reef habitats surrounding Qilian Yu Island (112°14′–112°21′E, 16°55′–17°00′N), in the Xuande group (**Supplementary Table S1** and **Supplementary Figure S1**). The ocean hydrological characteristics are relatively stable here, and the coral reef ecosystems are largely not affected by human activities and terrestrial runoff (Ye, 1996; Huang et al., 2008). The coral reef in Paracel Island typically distributes in irregular block, and some even suffering coral bleaching, therefore, all the sampling sites were chosen among the coral reef with high biology diversity. The sediment samples were collected in 6–14 m water depth within the coral reef, according to their actual depth in different location and duplicate samples were collected from each sampling site. The coral reef sediments of sampling sites consist of sandy calcareous sands. The temperature and salinity of Qilian Yu during sampling period were relatively stable. The top 1 cm of sediment was discarded, and the underlying 2–5 cm depth of sediment was retained according to pervious study. The sediment (200 ml volume) and seawater (150 ml) samples were collected into 500-ml plastic bottles, and MMPs in the samples were magnetically enriched from the samples on board the research vessel immediately following sampling. This was achieved by placing the south poles of two 0.37 T permanent magnets against opposite outer surfaces of the bottles, adjacent to the water–sediment interface (Flies et al., 2005). After 30 min, approximately 1 ml of the water containing brown–blackish spots that formed in the bottles adjacent to the magnets was withdrawn into a 1.5-ml centrifuge tube. The bacteria in the withdrawn sample were inspected using the hanging drop method and counted (Schüler, 2002; Flies et al., 2005). The enriched MMPs were magnetically concentrated and purified using the tube-track method, as previously described (Zhou et al., 2013). Purified MMPs were used to make electron microscopy observations. Following observations, the samples were stored under dim light at 16°C on the research vessel for transport to the laboratory for micromanipulation and phylogenetic analysis.

### Optical and Electron Microscopy

Multicellular magnetotactic prokaryotes were observed in the hanging drop assay (Schüler, 2002; Flies et al., 2005) using an Olympus BX51 phase contrast microscope equipped with a Canon 700D camera. The concentrated samples removed from sediment were left into the magnetic field for approximately 30 min before counting. The entire water drop border was recorded, both north-seeking and south-seeking MMPs were counted. For transmission electron microscopy (TEM) observations, purified samples were adsorbed onto 200 mesh Formvar carbon copper grids (Beijing Zhong Jing Ke Yi Technology Co. Ltd.), and examined using a Hitachi HT7700 transmission electron microscope operating at 100 kV. The composition of magnetosomes and inclusions was investigated using high resolution TEM (HRTEM; Joel JEM-2100 microscope, operating at 200 kV) and energy dispersive X-ray spectroscopy (EDXS). To investigate the surface morphology of the MMPs, the cells were fixed with glutaraldehyde at the final concentration of 1.25% and stored at 4°C during the voyage, and then transferred onto 0.2-μm nuclepore polycarbonate (Whatman, Britain) *via* extraction filtration when came back to laboratory. Afterward, the samples were dehydrated with ethanol and isoamyl acetate. Properly dried and gold coated samples were examined using scanning electron microscopy (SEM) using a KYKY-2800B scanning electron microscope (KYKY Technology Development Ltd., China) operating at 25 kV.

### Physical and Chemical Characteristics of Sediments

For granulometric analysis, the sediments were dried at 80°C for 3 h then sieved sequentially through 4, 2, 1, 0.5, 0.25, 0.125, 0.1, and 0.063 mm meshes. The fractions were weighed and examined using an Olympus SZX16 stereo microscope. Measurements were performed on the samples from all 11 stations. The salinity and pH were determined using a hand held refractometer (YW100; Chenhua, Chengdu, China) and a benchtop pH/temperature meter (JENCO 6173; Shanghai, China), respectively.

### Phylogenetic Analysis

For phylogenetic analysis of MMPs from the coral reef habitats, we combined micromanipulation with WGA, followed by polymerase chain reaction (PCR) amplification of the 16S rRNA gene. The procedures of using micromanipulation for identification of MTB and MMPs genome sequencing have been described previously (Jogler et al., 2011; Kolinko et al., 2012; Zhang et al., 2014; Chen et al., 2015, 2016; Liu et al., 2017, 2018; Teng et al., 2017) and WGA of the cells of MMPs was carried out according to the provided guidelines of the illustra^TM^ Single Cell GenomiPhi^TM^ DNA Amplification Kit (GE29-1080-39; Sigma, United States), with an amplification time of 2.5 h. The bacteria-specific primers 27f and 1492r (Sangon Biotech, Shanghai, China) were used for the PCR (Frank et al., 2008), which was performed using a Mastercycler (Eppendorf, German). The resulting PCR products were cloned into the pMD18-T vector (Takara, Dalian, China) and transformed into competent *E. coli* TOP10 cells. The clones were randomly selected for sequencing, which was carried out by Nanjing Genscript Biotechnology (Nanjing, China) (Teng et al., 2017).

The 16S rRNA gene sequences of MMPs obtained in this study were analyzed using the BLAST search program^1^. All the sequences were aligned using CLUSTAL W multiple alignment software, and sequence identities were calculated using the BIOEDIT software. A phylogenetic tree was constructed using the neighbor-joining method in MEGA 6.0, and bootstrap values were calculated from 1000 replicates (Teng et al., 2017). The sequences were submitted in the GenBank database under accession numbers KY21895–KY21900.

## Results

### Physical and Chemical Characteristics of Sediments

Sediments from the coral reef habitats of the Paracel Islands were sorted into gravel, sand, and silt, and analyzed (**Supplementary Figure S2**). The presence of angular grains suggested that the environment is generally hydrodynamically calm. The grain size varied from <0.063 to >4 mm, with sand in the range 2–0.063 mm (arenite granulometric class) comprising the largest proportion. The sediments contained large quantities of biological detritus including shells, fragment of gastropods, coral detritus, and sea urchin spicules. The pH was 7.62–7.89 and the salinity was 37‰.

### Morphological Characterization of MMPs and Their Magnetosomes

The MMPs were investigated using optical microscopy following magnetic enrichment. The s-MMP morphotype was found in samples from nine stations (average abundance: 0.25 ind./cm^3^) and e-MMPs were found at seven stations (average abundance: 0.943 ind./cm^3^). The maximum abundance of MMPs found was 6 ind./cm^3^ (**Supplementary Table S1**). Based on the observation under optical microscopy, two out of nine stations, C11 and C14, were observed containing both North- and South-seeking s-MMPs, with the ratio of 5:1 and 25:1, respectively. While, only one station, C11, contained both North- and South-seeking e-MMPs, with the ratio of 5:1.

**FIGURE 1:**
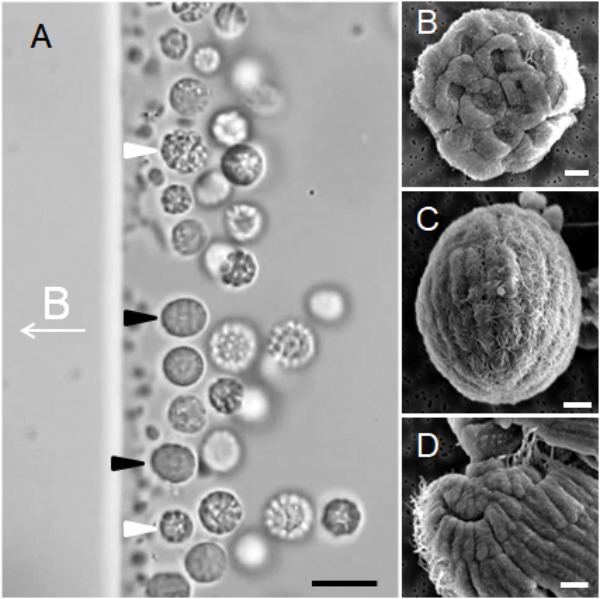
Occurrence and morphology of MMPs. **(A)** Differential interference contrast image showing MMPs aligned to the magnetic field (the direction of the applied magnetic field is indicated at the middle left, B symbol for magnetic induction intensity) and accumulating at the north pole of the droplet. The white and black arrows indicate s-MMPs and e-MMPs, respectively. **(B)** SEM images showing an integrated s-MMP cell. **(C)** SEM images showing integrated e-MMP cells that have formed five interlaced circles, with flagella evident on the surface. **(D)** SEM images showing cracked e-MMP with triangle and rectangle-shaped cells. Scale bars = 10 μm in panel **(A)** and 1 μm in the other panels.

Optical microscopy showed that the e-MMPs were 7.47 ± 1.65 μm × 6.04 ± 1.21 μm (*n* = 177) in size, and the average diameter of the s-MMPs was 5.87 ± 1.37 μm (*n* = 166) (**Figure 1A**). SEM analysis revealed differing surface structures associated with s-MMPs and e-MMPs, which were flagellated on their outer surfaces (**Figure 1C**). Each s-MMP was composed of approximately 30 ovoid cells arranged in a helix, and each cell was 1.67 ± 0.31 mm (*n* = 12) (**Figure 1B**). Individual e-MMPs were composed of up to 100 cells arranged in 5–7 interlaced circles that formed grooves at the joint areas. The cells forming the top one to two cell circles at the poles of the e-MMPs appeared to be triangle-shaped, whereas those in the middle were rectangle-shaped (**Figure 1D**). Each cell averaged 0.51 ± 0.08 μm (*n* = 75) in width, and were 0.78 ± 0.33 μm (*n* = 18) and 1.49 ± 0.32 μm (*n* = 17) in length for cells in the polar and middle cell circles, respectively. These observations resemble the reports of MMPs previously (**Figure 1C**; Zhou et al., 2012; Chen et al., 2015, 2016).

**FIGURE 2:**
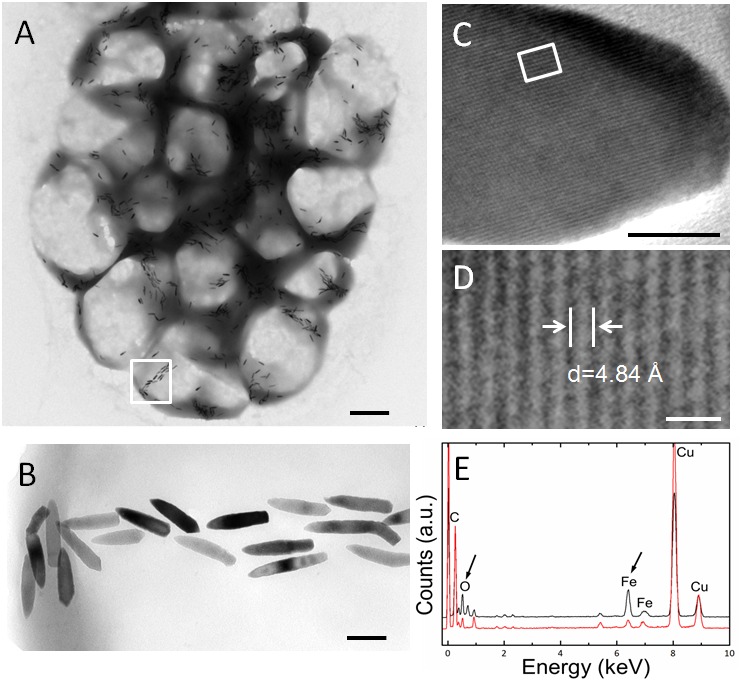
Characteristics of magnetosomes in s-MMPs. **(A)** Overview TEM images of s-MMPs containing bullet-shaped magnetosomes. **(B)** Magnification of a selected area from panel **(A)**, showing the bullet-shaped magnetosomes. **(C,D)** HRTEM analysis identification of the magnetite (Fe_3_O_4_) in the bullet-shaped magnetosomes. **(E)** EDXS analysis showing the spectrum of magnetite (Fe_3_O_4_) in the bullet-shaped magnetosomes. The black and red lines represent spectrum from magnetosome and cytoplasm, respectively. The black arrows indicate peaks of iron and oxygen. Scale bars = 1 μm in **(A)**, 100 nm in **(B)**, 10 nm in **(C)**, and 1 nm in **(D)**.

**FIGURE 3:**
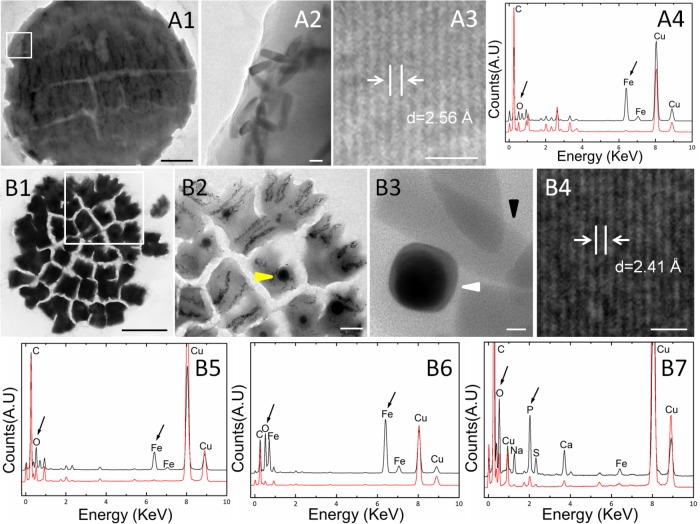
Characteristics of magnetosomes in e-MMPs. **(A)** MMPs containing only bullet-shaped magnetosomes. Overall **(A1)** and magnified **(A2)** TEM views showing the bullet-shaped magnetosomes. HRTEM **(A3)** and EDXS **(A4)** spectra for the magnetite (Fe_3_O_4_) in the bullet-shaped magnetosomes. **(B)** MMPs containing both bullet-shaped and rectangular magnetosomes. Overall **(B1)** and magnified TEM views showing inclusion bodies [**(B2)**, yellow arrow] and bullet-shaped magnetosomes [**(B3)**, black arrow] clustered with octahedral magnetosomes [**(B3)**, white arrow]. HRTEM **(B4)** and EDXS **(B5)** analysis showing the spectrum of magnetite (Fe_3_O_4_) in the bullet-shaped magnetosomes. EDXS analysis of octahedral magnetosomes **(B6)** and polyphosphate in the inclusions **(B7)** respectively. Scale bars = 2 μm in **(A1)**, 50 nm in **(A2)**, 1 nm in **(A3,B4)**, 5 μm in **(B1)**, 1 μm in **(B2)**, and 10 nm in **(B3)**.

Transmission electron microscopy showed that the s-MMPs from the Paracel Islands synthesized only bullet-shaped magnetosomes in different shape factors, which were arranged in chains or clusters (**Figure 2A**). The amount of magnetosomes per s-MMP varied from 233 to 1189, and each cell contained 35 ± 17 magnetosomes (*n* = 38). The bullet-shaped magnetosomes were 139.4 ± 36.3 nm × 39.2 ± 3.5 nm (*n* = 149), and the width/length ratio was 0.30 ± 0.10 (*n* = 149) (**Figure 2B**). HRTEM and EDXS analyses indicated that the magnetosomes were composed of magnetite (Fe_3_O_4_) crystals (**Figures 2C–E**). TEM-based observations revealed that some of the e-MMPs contained only bullet-shaped magnetosomes (**Figures 3A1,A2**), while others contained both bullet-shaped and octahedral crystals (**Figures 3B1–B3**). In integrated e-MMPs, the magnetosomes in the middle cell circles were clustered linearly, and ran approximately parallel to the long axis of the e-MMP, and crossed at bipolar. The magnetosomes of the e-MMPs from the coral reef habitats were poorly organized compared with those of e-MMPs from the intertidal zone of Lake Yuehu in Rongcheng Bay (China) (Chen et al., 2015), and from Drummond Island in the South China Sea (Chen et al., 2016). Well-ordered magnetosome chains optimize the magnetic moment of the entire aggregate (Wenter et al., 2009). The magnetosomes of disintegrated e-MMPs were arranged into several parallel chains in every planar cell. The number of magnetosomes in each e-MMP varied from 2421 to 7486, and the average number of magnetosomes per cell was 45 ± 20 (*n* = 39). The bullet-shaped magnetosomes were 134 ± 23 nm in length and 40 ± 4 nm (*n* = 182) in width, and the breadth/length ratio was 0.30 ± 0.06 (*n* = 182). The octahedral crystals were approximately 40 nm in size (**Figure 3B3**). HRTEM and EDXS analyses revealed that both types of magnetosome were composed of magnetite (Fe_3_O_4_) (**Figures 3A3,A4,B4–B6**). EDXS analyses showed that the inclusions in the e-MMPs contained large quantities of phosphorus and oxygen, possibly in the form of polyphosphate (**Figure 3B7**).

### Phylogenetic Analysis

**FIGURE 4:**
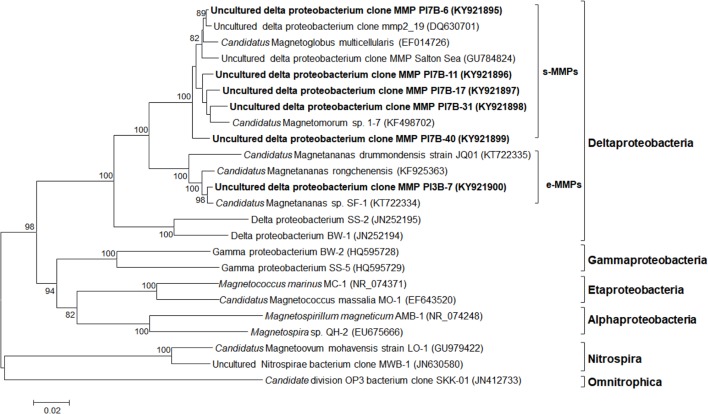
Phylogenetic tree of MMPs based on 16S rRNA gene sequences. The tree was constructed based on neighbor-joining analysis. The sequences determined in this study are shown in bold. GenBank accession numbers are indicated in parentheses. Numbers at nodes represent bootstrap values and are default less than 80. Bars = 0.02 substitutions/nucleotide position.

Two microsorted samples containing 11 s-MMPs and 1 e-MMP, respectively, were obtained from Paracel Islands coral reef sediment samples and were subjected to 16S rRNA gene analysis. The extracted genomic DNA was amplified using the multiple displacement amplification (MDA) method, and then the 16S rRNA gene was amplified and cloned. In total, 36 and 20 randomly chosen clones of s-MMPs and the e-MMP were sequenced, respectively. Among the 36 s-MMP sequences, a total of 5 OTUs (<97% sequence identity) were obtained. The 18 e-MMP sequences (2 of the 20 were derived from false positive clones) shared at least 99.2% sequence identity. Phylogenetic analysis (**Figure 4**) of the 16S rRNA gene sequences revealed that all MMPs were affiliated with the *Deltaproteobacteria*. The s-MMPs clustered into a single clade that was separate from the clade of the e-MMPs. The 5 species of s-MMPs were named as the uncultured *Deltaproteobacteria* MMP clones PI7B-6 (KY21895; 16 sequences), PI7B-11 (KY21896; 1 sequence), PI7B-17 (KY21897; 1 sequence), PI7B-31 (KY21898; 15 sequences), and PI7B-40 (KY21899; 3 sequences), and the e-MMP species was designated as uncultured *Deltaproteobacteria* MMP clone PI3B-7 (KY21900).

Based on a BLAST search of the GenBank database, the uncultured *Deltaproteobacteria* clone PI7B-6 was most closely related (98.9% sequence identity) to the uncultured *Deltaproteobacteria* MMP clone mmp2_19 (DQ630701) from the Sippewissett salt marsh in Falmouth (United States) (Simmons and Edwards, 2007), and to PI7B-11 (96% sequence identity). *Candidatus* Magnetomorum sp. 1-7 (KF498702), from Lake Yuehu in Rongcheng Bay of China (Zhang et al., 2014), showed the highest level of identity with the uncultured *Deltaproteobacteria* MMP clones PI7B-17 (97% sequence identity), PI7B-31 (97.1% sequence identity), and PI7B-40 (96.4% sequence identity). The uncultured *Deltaproteobacteria* e-MMP clone PI3B-7 from the Paracel Islands shared 99 and 98.2% sequence identity with *Candidatus* Magnetananas sp. SF-1 (KT722334) from Six-Fours-les-Plages (France) (Chen et al., 2016) and *Candidatus* Magnetananas rongchenensis (KF925363) from Lake Yuehu in the Yellow Sea, respectively, which indicated that they share the same phylotype despite being highly geographically separated. The results indicate that two new species of s-MMP (PI7B-11 and PI7B-40) were amongst those identified from the Paracel Islands.

## Discussion

To increase knowledge of MMP communities in coral reef habitats, we made an investigation on the diversity of MMPs from Qilian Yu Islands. These islands are part of the Paracel Islands, have a similar natural environment to that of the entire Paracel Islands. The Paracel Islands are located in the central tropics with a year-round high temperatures and the annual average air temperature of 26–27°C. In this study, we found MMPs at 9 of 11 sampling stations that encompassed most of the area having reef coral habitat at Qilian Yu Islands. The study indicated that two MMP morphotypes, s-MMPs and e-MMPs, are widely distributed in coral reef habitats of the Paracel Islands. Among the two morphotypes, we identified one species of e-MMP and five species of s-MMP; the latter MMPs included two new species. These findings expand and highlight the known distribution and diversity of MMPs, and comprise the first report of MMPs from coral reef habitats. Our study also shows that s-MMPs and e-MMPs were widely distributed, and not just limited to the intertidal zone (Lefèvre et al., 2007; Chen et al., 2015, 2016).

In previous studies, the sampling sediment always contained gray–black layers due to changes in sulfur content with depth (Wenter et al., 2009; Zhang et al., 2014; Chen et al., 2015; Du et al., 2015; Liu et al., 2018). It is reported that greigite biomineralization in some MTB (Simmons et al., 2004; Simmons and Edwards, 2007; Lefèvre et al., 2011, 2013; Chen et al., 2015) may reflect their sulfate-reducing lifestyle adapted to more reducing conditions. In this study, gray or black layers were not observed during sampling, and the sediment was typical calcareous sand in white. Therefore, the oxidation potential of the sampling sites in coral reef habitats may be higher compared with those in gray–black layers theoretically. And the fact that no greigite crystals were found in our study also verified a less reducing condition in coral reef habitats. Previously studies also demonstrated that dissolved O_2_ can penetrate 15–30 cm into the Checker Reef sediments, depending on wave action and sediment permeability (Haberstroh and Sansone, 1999; Falter and Sansone, 2000a,b). The less reducing environment may imply higher dissolved oxygen concentration, thereby, may indicate the coral reef ecosystem in Qilian Yu is healthy and of good quality. It has been demonstrated that salinity is a key determinant of MTB abundance and biogeography (Martins et al., 2009, 2012; Lin et al., 2013; Du et al., 2015) as well as coral reef fertilization and embryo development (Humphrey et al., 2008). MMPs lost cells shape and disaggregated (Martins et al., 2009), and coral reef embryos were observed to develop abnormal and no fertilization occurred (Humphrey et al., 2008) when undergoing low salinity conditions. Based on the investigation and analysis of environmental factors of coral reef habitats in the tropical ocean (Guan et al., 2015), the salinity and pH of seawater in Paracel Islands where the MMPs were observed are within the tolerance limits of coral. Hence, MMPs may be used as indicator species in future monitoring the evolution and water quality for coral reef ecosystem.

Coral reefs are among the most productive and biologically diverse habitats (Moberg and Folke, 1999; Garren and Azam, 2012), are host to an extraordinary variety of marine plants and animals, and constitute one of the most spectacular ecosystems on earth (Spalding et al., 2001). In the tropical surface waters surrounding coral reefs, the micronutrient iron is typically found at low concentration (Gordon et al., 1997; Blain et al., 2008). Thus, iron has been proposed to limit primary productivity (Ferrier-Pages et al., 2001; Rodriguez et al., 2016) due to its essential role in most metabolic reactions, such as photosynthesis and microbial N_2_ fixation (Price, 1968). Owing to the anthropogenic activity, such as shipwreck (Sandin et al., 2008; Work et al., 2008), Fe element has been continuously released into the surrounding waters of coral reef from the source of iron. The Fe supplementation supports the overgrowth of phytoplankton and heterotrophic microbes, increases the respiration rates significantly (Radecker et al., 2017), and resulting in black reefs with a dramatic loss of corals and crustose coralline algae cover (Schroeder et al., 2008; Work et al., 2008). Iron enrichment can also cause the dramatic decrease of microbial nitrogen fixation and may exacerbate the limitation of other nutrients (Kelly et al., 2012), creating a negative feedback loop (Radecker et al., 2017). Iron enrichment turned the oligotrophic waters into eutrophication and resulting in a decrease in pH and aragonite saturation state (Ω_arag_) as well as depletion in dissolved oxygen (Xu et al., 2016). Ω_arag_ represents the effect of ocean acidification on biologically mediated CaCO_3_ precipitation (Waldbusser and Salisbury, 2014), and several studies indicate that coral calcification decreases with declining Ω_arag_ (Chris et al., 2000; Marubini et al., 2008). Together, redundant iron poses significant threats to coral reefs in iron-limited regions.

Magnetotactic bacteria, especially MMPs is typically considered as the pioneer in biomineralization Fe to biosynthesize magnetite and greigite. MTB may accumulate up to 100 times more iron than other non-magnetotactic heterotrophic bacteria (Schüler and Baeuerlein, 1996). It is estimated that the magnetosomes production varies from 2.6 to 41.7 mg/L (Araujo et al., 2015) in unicellular MTB and 1.16 × 10^-12^ g per *Ca.* M. multicellularis (Martins et al., 2009). According to the size (82 nm in diameter) and amount (905 per microorganism) of magnetosomes in *Ca.* M. multicellularis (Martins et al., 2009), the production of magnetosomes in MMPs in this study may equal to that of *Ca.* M. multicellularis. It has been identified that ciliates prey on MMPs and magnetosomes within their acidic vacuoles can be dissolved in a more soluble form (Martins et al., 2007; Garren and Azam, 2012), which make iron from magnetosome able to participate in the metabolic process (Martins et al., 2009) and biogeochemical cycles among the coral reef ecosystem. Therefore, MMPs may act as reservoir of redundant iron to maintain the iron concentration under reasonable level and play a crucial role in iron cycling in such oligotrophic environment.

A healthy coral reef functions as a finely tuned microbially driven system that excels at efficient uptake and recycling of nutrients in oligotrophic waters (Garren and Azam, 2012). Diverse microorganisms in these ecosystems exert a significant influence on biogeochemical and ecological processes (Wild et al., 2006; Mayer and Wild, 2010; Mouchka et al., 2010; Tremblay et al., 2011). It has been demonstrated that the microbial communities in the reef sediment play an important role in benthic-pelagic coupling of nitrogen cycling (Gaidos et al., 2011). And all benthic environments associated with coral reef that have been examined, were found to have nitrogen fixation (Larkum et al., 1988; Shashar et al., 1994; Casareto et al., 2008; Charpy et al., 2010). Most MTB strains also exhibit nitrogenase activity and contain a full suite of *nif* genes in their genomes (Bazylinski and Blakemore, 1983; Bazylinski et al., 2000; Schübbe et al., 2009). Thus, we can speculate the MMPs take part in the nitrogen cycling in coral reef ecosystem despite that the axenic culture and complete genome of MMPs are not yet available.

It is pointed out that the swimming polarity of s-MMPs can change during an isolation process in the presence of high magnetic fields and magnetic field gradients and the swimming polarity reversal depends on the magnetic moment intensity of MMPs (de Melo and Acosta-Avalos, 2017). In this study, a small proportion of south-seeking organisms were also observed in two samples. Since the magnetic fields applied from magnets are equal between all the samples, the magnetic moment intensity of south-seeking MMPs must be higher than the north-seeking ones. The s-MMPs have been reported to crystallize iron sulfide greigite magnetosomes having irregular shapes, including roughly equi-dimensional or flake shapes (Farina et al., 1983, 1990; Mann et al., 1990; Keim et al., 2004a; Abreu et al., 2007; Wenter et al., 2009), bullet-shaped magnetite (Zhou et al., 2011, 2013) or greigite magnetosomes (Wenter et al., 2009), or both equi-dimensional greigite and bullet-shaped magnetite magnetosomes (Lins et al., 2007; Zhang et al., 2014). Bullet-shaped magnetosomes are relatively rarer than irregular magnetosomes, and greigite crystals are more common in s-MMPs. Two phenotypes of s-MMP recently identified from the Yellow Sea area (one from Lake Yuehu and the other from Huiquan Bay) which are similar in size, can produce only bullet-shaped magnetite magnetosomes of 92 nm × 30 nm size (Zhou et al., 2011, 2013). The s-MMPs from Huiquan Bay are composed of approximately 15–30 cells that can synthesize bullet-shaped magnetites in chains or clusters, while those from Lake Yuehu are usually composed of 10–16 ovoid cells which can biomineralize bullet-shaped magnetite crystals in highly organized parallel chains. While another s-MMPs identified from Lake Yuehu usually contain 16–32 ovoid cells and can synthesize both bullet-shaped magnetosomes (80 nm × 34 nm in size) and irregular magnetosomes (64 nm × 53 nm in size), which were also arranged in chains or clusters (Zhang et al., 2014). In the present study, the magnetosomes in s-MMPs from coral reef habitats of the Paracel Islands were all bullet-shaped magnetite crystals approximately 139 nm × 39 nm in size, which is larger than those previously reported (Zhou et al., 2011, 2013). The e-MMPs predominantly contained bullet-shaped magnetite crystals (Zhou et al., 2012; Chen et al., 2015, 2016), whereas one type of e-MMP from Lake Yuehu had both bullet-shaped magnetite and irregular greigite magnetosomes (Chen et al., 2015). Interestingly, the e-MMPs in this study were able to synthesize magnetite crystals in bullet and octahedral shapes. Similar observations have been previously been made for unicellular MTB (Li et al., 2015). Based on Li et al. (2015), octahedral magnetosomes in e-MMPs in our study may be nascent crystal with cubo-octahedron shape in the initial isotropic growth stage of biomineralization of bullet-shaped magnetite magnetosomes, since the cubo-octahedron crystals are typically about 35–40 nm, similar to the octahedral magnetosomes in our study (Li et al., 2015). All MMPs found in this study contained only magnetite magnetosomes, which may be related to the environment of coral reef habitats, indicating that further analysis of the potential role of environmental factors at the Paracel Islands in magnetosome formation is needed.

## Author Contributions

WZ, HP, HH, L-FW, and TX designed the research. ZT, YZ, and JX prepared samples. ZT, WZ, and HP carried out the experiments. ZT and WZ prepared the manuscript.

## Conflict of Interest Statement

The authors declare that the research was conducted in the absence of any commercial or financial relationships that could be construed as a potential conflict of interest. The reviewer FA declared a past co-authorship with the authors to the handling Editor.

## Footnotes

1 http://www.ncbi.nlm.nih.gov/BLAST/
