# Supplementary material for: Diversity and Characterization of Multicellular Magnetotactic Prokaryotes From Coral Reef Habitats of the Paracel Islands, South China Sea
Source: Front Microbiol. 2018 Sep 11;9:2135. doi: 10.3389/fmicb.2018.02135 (PMC6142882; doi:10.3389/fmicb.2018.02135)

**Supplemental information**

# Diversity and characterization of multicellular magnetotactic prokaryotes from coral reef habitats of the Paracel Islands, South China Sea

Zhaojie Teng ^1, 2, 3, 5^, Yuyang Zhang ^4^, Wenyan Zhang ^1, 2, 3, 7 *^, Hongmiao Pan ^1, 2, 3, 7 *^, Jianhong Xu ^1^, Hui Huang ^4^, Tian Xiao ^1, 2, 3, 7^, Long-Fei Wu ^6, 7^

^1^ CAS Key Laboratory of Marine Ecology and Environmental Sciences, Institute of Oceanology, Chinese Academy of Sciences, Qingdao, China

^2^ Laboratory for Marine Ecology and Environmental Science, Qingdao National Laboratory for Marine Science and Technology, Qingdao, China

^3^ Center for Ocean Mega-Science, Chinese Academy of Sciences, Qingdao, China

^4^ Key Laboratory of Marine Bio-resources Sustainable Utilization, South China Sea Institute of Oceanology, Chinese Academy of Sciences, Guangzhou, China

^5^ University of Chinese Academy of Sciences, Beijing, China

^6^ Aix Marseille University, CNRS, LCB, Marseille, France

^7^ International Associated Laboratory of Evolution and Development of Magnetotactic Multicellular Organisms (LIA-MagMC), CNRS-CAS, Marseille-Qingdao, France-China

**For correspondence,** Wenyan Zhang, E-mail: zhangwy@qdio.ac.cn; Hongmiao Pan, E-mail: panhongmiao@qdio.ac.cn.

**Table S1. Information of the sampling sites and MTB abundance.**

| **Site** | **Longitude** | **Latitude** | **Depth (m)** | **Abundance (ind./cm^3^)** | | |
| --- | --- | --- | --- | --- | --- | --- |
|  |  |  |  | **s-MMPs** | **e-MMPs** | **Total** |
| C1 | 112°17′35″ | 16°58′22″ | 9 | 0.02 | 0 | 0.02 |
| C2 | 112°16′06″ | 16°58′14″ | 9 | 1 | 1 | 2 |
| C3 | 112°14′36″ | 16°58′13″ | 9 | 0.2 | 1.04 | 1.24 |
| C6 | 112°16′02″ | 16°59′34″ | 11 | 0.2 | 6.02 | 6.22 |
| C7 | 112°20′08″ | 16°57′17″ | 10 | 0.4 | 0.2 | 0.6 |
| C8 | 112°20′29″ | 16°55′08″ | 6 | 0.04 | 1.64 | 1.68 |
| C9 | 112°20′00″ | 16°56′06″ | 10 | 0 | 0 | 0 |
| C10 | 112°19′25″ | 16°57′06″ | 6 | 0 | 0 | 0 |
| C11 | 112°18′15″ | 16°57′47″ | 6 | 0.24 | 1.2 | 1.44 |
| C13 | 112°16′28″ | 16°58′07″ | 14 | 0.54 | 0 | 0.54 |
| C14 | 112°16′29″ | 16°58′07″ | 10 | 0.04 | 0.02 | 0.06 |

**Fig S1. The Paracel Islands location.**

The Paracel Islands, with sampling sites marked by red crosses (Modified base on website: www.nhjd.net).

**
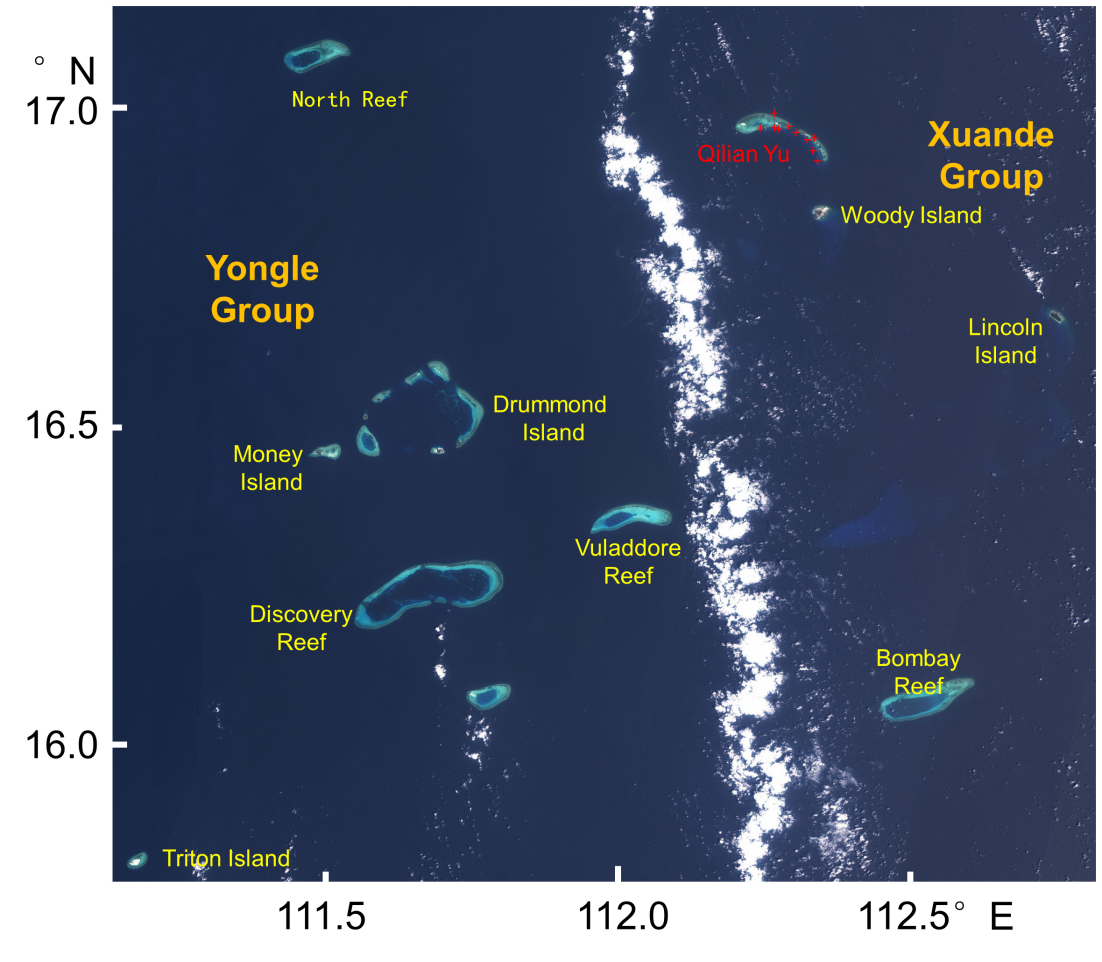
**

**Fig S2. Differential interference contrast images and histograms of the grain size distribution of sediment samples from the Paracel Islands.**

Boulderet: 64~4 mm. Fine gravel: 4~2 mm. Very coarse sand: 2~1 mm. Coarse sand: 1~0.5 mm. Medium sand: 0.5~0.25 mm. Fine sand: 0.25~0.125 mm. Very fine sand-1: 0.125~0.1 mm. Very fine sand-2: 0.1~0.063 mm. Silt: <0.063 mm. Scale bars = 2 mm.


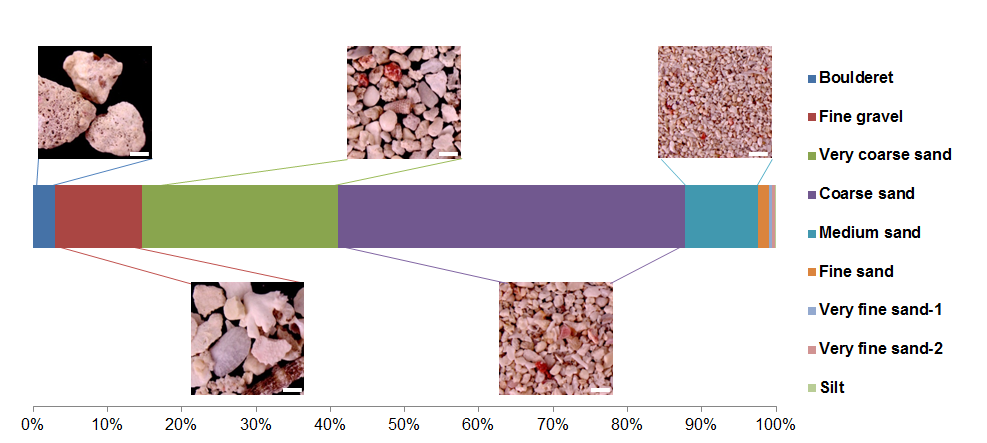

Supplement: Supplementary file 1 [file Data_Sheet_1.docx]
